# New CRISPR Tools to Correct Pathogenic Mutations in Usher Syndrome

**DOI:** 10.3390/ijms231911669

**Published:** 2022-10-01

**Authors:** Lauren Major, Michelle E. McClements, Robert E. MacLaren

**Affiliations:** 1 Laboratory of Ophthalmology, Nuffield Department of Clinical Neurosciences & NIHR Oxford Biomedical Research Centre, University of Oxford, Oxford OX3 9DU, UK; 2 Oxford Eye Hospital, Oxford University Hospitals NHS Foundation Trust, Oxford OX3 9DU, UK

**Keywords:** CRISPR/Cas9, base-editing, prime-editing, Usher Syndrome, genome engineering, gene therapy

## Abstract

Inherited retinal degenerations are a leading cause of blindness in the UK. Significant advances have been made to tackle this issue in recent years, with a pioneering FDA approved gene therapy treatment (Luxturna^®^), which targets a loss of function mutation in the *RPE65* gene. However, there remain notable shortcomings to this form of gene replacement therapy. In particular, the lack of viability for gene sequences exceeding the 4.7 kb adeno-associated virus (AAV) packaging limit or for toxic gain of function mutations. The *USH2A* gene at ~15.7 kb for instance is too large for AAV delivery: a safe and effective vehicle capable of transducing photoreceptor cells for gene replacement therapy. Usher Syndrome is a clinically and genetically heterogenous deaf-blindness syndrome with autosomal recessive inheritance. The *USH2A* gene encodes the protein usherin, which localises to the photoreceptor cilium and cochlear hair cells. Mutations in the *USH2A* gene cause Usher Syndrome type II (USH2), which is the most common subtype of Usher Syndrome and the focus of this review. To date, researchers have been unable to create an efficient, safe editing tool that is small enough to fit inside a single AAV vector for delivery into human cells. This article reviews the potential of CRISPR technology, derived from bacterial defence mechanisms, to overcome these challenges; delivering tools to precisely edit and correct small insertions, deletions and base transitions in *USH2A* without the need to deliver the full-length gene. Such an ultra-compact therapy could make strides in combating a significant cause of blindness in young people.

## 1. Introduction

Usher syndrome was first described by the Scottish ophthalmologist Charles Usher in 1914. It is a deaf-blindness syndrome encompassing a triad of sensory dysfunction: retinitis pigmentosa, congenital auditory deficits and variable vestibular function. Patients are placed into one of three categories (type I, II or III) based on the severity of their phenotype. Type I Usher syndrome is the most severe form: profound deafness is usually present from birth and sight loss occurs early in development [1]. Type I patients also suffer from impaired balance and delayed motor development [2]. Type II Usher Syndrome (USH2) manifests as a milder early onset hearing loss and sight loss progresses during adolescence [3]. Balance is typically unaffected. Type III Usher Syndrome is the rarest variant, more common in the Finnish population due to a founder effect [4]. Night vision starts to deteriorate around puberty and hearing loss develops in late childhood. Vestibular function is variable [1]. Different causative genes have been identified and allocated to each category (Table 1).

**Figure 1:**
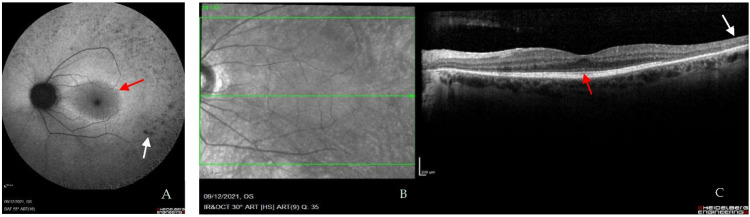
Example images of a patient with Usher (*USH2A*) syndrome. (**A**) Autofluorescence images show a ring of hypo-autofluorescence (red arrow) around the macula, a classic presentation of ciliopathy. The peripheral retina shows pigmentary changes (white arrow). The ring indicates loss of the photoreceptor outer segments and outside of the ring there is loss of rod photoreceptor cells. (**B**) Optical coherence tomography (OCT) images from the same patient showing an optical section through the retina (Central green arrow indicates position of horizontal section). (**C**) There is thinning of the peripheral retina (white arrow) and some loss of the outer segments. Intact outer segments are indicated by the red arrow. Scale bar 200 µm. Images from Oxford Eye Hospital, Oxford University Hospitals NHS Foundation Trust, Oxford.

Type II Usher patients represent about 50% of Usher cases [5]. To date, three genes have been ascribed to USH2 (2A, C and D), of which subtype 2A is the commonest form, comprising approximately 80% of USH2 patients. Usher 2A is an autosomal recessive ciliopathy (Figure 1) that leads to a truncated usherin protein: this triggers progressive deterioration of photoreceptor function, commencing with the more susceptible rod cells [6]. Mutations in *USH2A* are markedly heterogenous, with 1339 presumed pathogenic variants reported in the LOVD Database (accessed on 29 November 2021). Nonetheless, some variants are more widespread in the population. These include two mutations (c.2299del and c.2276G>T) in exon 13 comprising around 15–31% and 8% of *USH2A* cases, respectively [7,8] and one in exon 61: the c.11864G>A (p. Trp3955*) mutation generates a premature termination codon and accounts for ~3–5% worldwide cases [7]. Due to their prevalence, these three mutations represent the focus of this review.

## 2. Current Approaches

To date, researchers have explored dual vector systems or suboptimal CRISPR tools that can fit within a single AAV vector to target Usher Syndrome. Tools outside of the CRISPR domain have also been investigated such as stem cell therapies and antisense oligonucleotides. However, all of these approaches remain problematic due to a combination of unwanted off target effects, lack of efficiency or limited applicability.

### 2.1. CRISPR/Cas System

**Figure 2:**
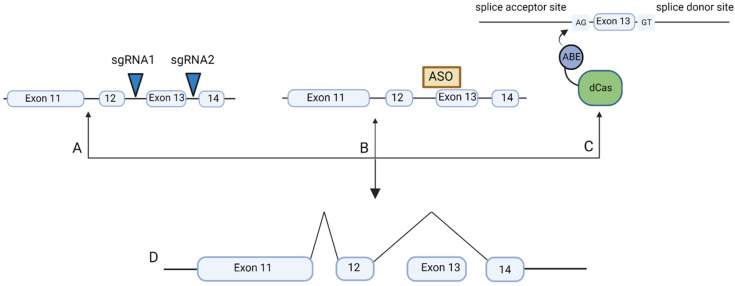
Gene therapy options for removing/skipping *USH2A* exon 13. (**A**) Excision of exon 13 with two sgRNAs that create DSB’s in the flanking introns, (**B**) Exon skipping of exon 13 mediated by antisense oligonucleotides, (**C**) Exon skipping mediated by base editing of the splice acceptor site (**D**) The final mRNA sequence excluding Exon 13 leading to a functional protein Created with BioRender.com (accessed on 22 August 2022).

The mutated domains could be excised from the *USH2A* gene using the CRISPR/Cas9 or Cas12 systems to induce double strand breaks at sites flanking the mutation (Figure 2A). An example of this is the EDIT-101 therapy targeting the *CEP290* gene that causes Leber Congenital Amaurosis. Dual *S. aureus* Cas9 guides cut the DNA either side of an intronic mutation that produces an aberrant splice donor site and truncated protein [9]. Ramifications to this approach include indel events during the double strand break repair pathway, off target effects and stochastic outcomes [10]. The intronic location of the *CEP290* mutation reduces the impact of unintended events, which would have increased significance if within the coding region. High fidelity variants are being generated to minimise off target effects. For example, HiFiCas9 contains altered amino acids in the Rec3 domain purposed for nucleotide recognition: the consequence is increased DNA/RNA heteroduplex specificity [11]. Moreover, it is possible to predict the location of double strand breaks and the error patterns of non-homologous end joining (NHEJ) mediated repair using a machine learning model [12] systems such as these could enable researchers to avoid unwanted off target frameshifts. CRISPR tools lacking fused editing enzymes have high potential for single AAV packaging. Nonetheless, the potential for inducing pathogenicity when targeting exonic *USH2A* mutations seems high where the intention is to retain a functional exon and CRISPR tools that avoid introduction of double strand breaks are preferable. Even when applied to intronic mutations, the safety of the CRISPR/Cas9 system is not as robust as it could be for therapeutic use; preclinical assessment of the EDIT 101 vector observed an off target event for one of its guides [13] and this figure could increase if the study were conducted over a longer time course.

### 2.2. Exon Skipping

Exon skipping is a mechanism of encouraging cellular machinery to jump over an exon containing a pathogenic mutation, directing the ribosome to translate proceeding exons. This offers great potential for averting a truncated usherin protein, so long as the transcript remains in frame, and offers some means of overcoming heterogeneity of mutations within exons.

Antisense oligonucleotides (nucleotides of length 15–30 that hybridise to mRNA) mask the splice acceptor site, prompting translation to proceed to the subsequent exon (Figure 2B). The antisense oligonucleotide Golodirsen has generated functional improvement in human clinical trials of Duchenne Muscular Dystrophy [14]. Golodirsen mediates skipping of exon 53 to restore the mRNA reading frame. The ProQR trial has enjoyed similar success by inducing skipping of exon 13 (length 642 bp, 214 codons) in the *USH2A* gene: the shortened usherin protein retained function, attributable to the repetitive structure of the gene [15]. The QR-421a treatment demonstrated restoration of electroretinogram function and usherin protein expression across in vivo models and has now progressed to clinical trials. A drawback to using antisense oligonucleotides for the treatment of inherited retinal disease is the requirement for repeated intravitreal injections: current trial data suggests the duration of effect is at least three months. CRISPR mediated splicing offers a more permanent treatment, reducing the expense, emotional impact and immunogenicity associated with repeated injections. The highly conserved consensus splice acceptor site (AG) could be modified with base editing [16] (Figure 2C). However, the adenine or cytidine base editor would need to be paired with a compact Cas protein for single vector packaging.

Excising exon 61 (±one exon either side) places the mRNA out of frame and therefore is not viable for the c.11864G>A *USH2A* mutation; however, exon skipping is a viable route for the mutations within exon 13. One option would be to place an antisense oligonucleotide over a cryptic splice site within the exon that would leave the mRNA in frame. Even so, there is no guarantee that a shortened usherin protein (excluding exon 61) would retain function: the degree of structural repetition observed at the start of the protein is lost towards the terminus. Exon skipping, whether mediated by CRISPR base editing or antisense oligonucleotides shows promise, however, its scope is somewhat limited for the reasons described.

### 2.3. Dual Vector Approaches

**Figure 3:**
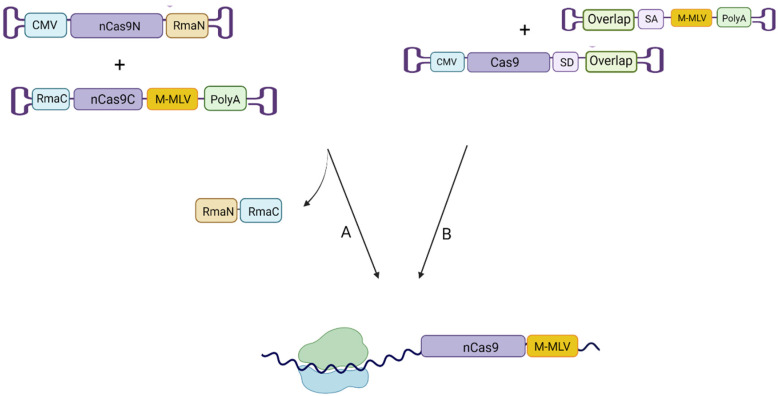
Example methods for dual vector delivery of large constructs. Delivery of a prime editing construct using dual vector approaches that employ (**A**) split Rma inteins or (**B**) hybrid approach. Created with BioRender.com (accessed on 22 August 2022).

Smaller genes such as *USH1C* fit within a single AAV vector and using gene supplementation, Pan et al. demonstrated a significant degree of auditory and vestibular rescue in a mouse model [17]. For larger genes, the 4.7 kb packaging limitation can be partially circumvented through employment of dual vectors. However, efficiency is compromised: Trapani et al. found that dual AAV8 vectors achieved only 6% of the retinal transduction rates found with the single vector [18]. A dual vector approach has been used to package the 6.7 kb cDNA of the *MYO7A* gene that leads to Usher Syndrome 1B [19]. The hybrid vector approach (Figure 3) was found to be most efficient: a region of sequence overlap enables recombination between transgenes with flanking splice acceptor and donor sites enabling removal of this DNA element following recombination. McClements et al. showed that the length of overlapping sequence between transgenes contributed to recombination efficiency, and further that dual AAV vectors could produce levels of *ABCA4* (6.8 kb) expression that provided a reduction in well-known markers of Stargardt disease in an *Abca4* knockout mouse model [20]. However, the size of the *USH2A* gene prohibits a similar dual vector approach; four to five vectors would be required in total and the recombination would be expected to be highly inefficient [21].

Rather than using dual vectors to deliver split gene fragments, dual vectors can be repurposed for split delivery of sophisticated CRISPR tools. Zhou et al. delivered a Cas protein and base editing enzyme within two separate vectors and induced recombination in vivo via attraction of ScFv antibody and GCN4 peptide fragments linked to the two separate components [22]. They were able to achieve significant correction of point mutations in phenylketonuria (*PKU*) mice, however a long-term study is needed to explore the potential for immunogenicity induced by the antibody and peptide fragments. The split intein method (Figure 3) relies on recombination of a divided Cas protein after transfection and Chen et al. demonstrated up to 25% editing in adult mouse retina; nonetheless there are similar concerns that the bacteria-derived intein protein could stimulate the innate immune system [23]. Liu et al. have shown that the Cas9 protein and reverse transcriptase can achieve comparable levels of editing when delivered in separate AAV vectors with no physical tether [24]. However, non-specific off target activity is a clear concern. A more promising strategy may be recombination of the full length transcript via the hybrid approach: trans-splicing carries reduced immunogenic risk and promises higher levels of efficiency [25]. Greater control could be achieved by leaving the splice acceptor site out of frame until splicing occurs to ensure the enzyme remains inactive until tethered. Nonetheless, the reduced efficiency associated with dual vector approaches is a persistent problem.

### 2.4. Stem Cell Treatments

Embryonic stem cells or induced pluripotent stem cells can be differentiated into retinal sheets and transplanted into the back of the eye [26]. To date this work has focused on rescue of retinal pigment epithelium in diseases such as Stargardts or age-related macular degeneration (AMD). However, Shirai et al. demonstrated improved light sensitivity and evidence of synaptic connections between graft and recipient cells in monkey models of retinal degeneration [27]. A considerable drawback to this approach is the tumorigenic potential of stem cell therapies [28].

Nonetheless, trials are ongoing including the ReNeuron trial, which injects human retinal progenitor cells (hRPC’s) beneath the retina. Researchers found that hRPC’s protect endogenous photoreceptors in a rodent model of retinal degeneration and that the rats showed significantly greater visual acuity when compared to rats receiving the control [29]. The therapy progressed to phase 2a clinical trial, with patients showing improvement in visual acuity, but not enough to reach FDA approved standards. The ReNeuron programme has now been paused due to surgical complications thought to be associated with injection of higher doses under the retina. No side effects related to the treatment itself have been noted, however, a longer term study is vital to properly assess the risk and predisposing factors involved in potential tumour development. Beam Therapeutics has combined base editing with patient derived haematopoietic stem cells to reactivate fetal haemoglobin in sickle cell disease and beta-thalassemia [30]. Phase 1/2 clinical trials have recently been approved by the FDA. Additionally, the jCyte trial is set to progress to phase 3 of clinical trial. Retinal progenitor cells are injected into the vitreous: the cells do not integrate into the retina but provide neurotrophic factors to the existing photoreceptors making this treatment most effective for patients identified early in the disease course [31]. A therapy that generates phenotypic improvement for patients further along in the disease progression would be preferable to one that carries risk of tumorigenesis and provides an incomplete and temporary halt to photoreceptor degeneration.

## 3. Ambition for Future Strategies

While showing promise, these existing strategies are not ideal for therapeutic use: dual vector approaches struggle to produce therapeutic levels of editing and often pose a risk of immunogenicity, whereas unmodified CRISPR/Cas constructs generate double stranded breaks in the DNA and carry considerable safety concerns; especially when mediating cuts within coding regions of DNA. Stem cell therapies pose a questionable risk of tumour development whereas exon skipping, although useful for certain mutations, lacks applicability where the gene is placed out of frame or where the skipped exon has a crucial role. Moreover, antisense oligonucleotides have a temporary effect, necessitating repeated injections. Progressing forwards, prime editing or base editing strategies offer superior specificity and can make precise single base edits. The current difficulty surrounds getting these constructs small enough to fit within a single AAV vector; a challenging task considering the unforgiving packaging limit of the AAV vector and the magnitude of the most effective CRISPR proteins. Two strategies are being explored in tandem: searching for new, smaller species of Cas proteins or base/prime editors derived from a myriad of existing or emerging organisms, in addition to attempts at truncating existing proteins. An efficient base editing or prime editing construct that can fit within a single AAV vector has real potential for therapeutic correction of many *USH2A* mutations.

**Evaluation of these approaches:**

### 3.1. Creation of a Smaller Base Editor

**Figure 4:**
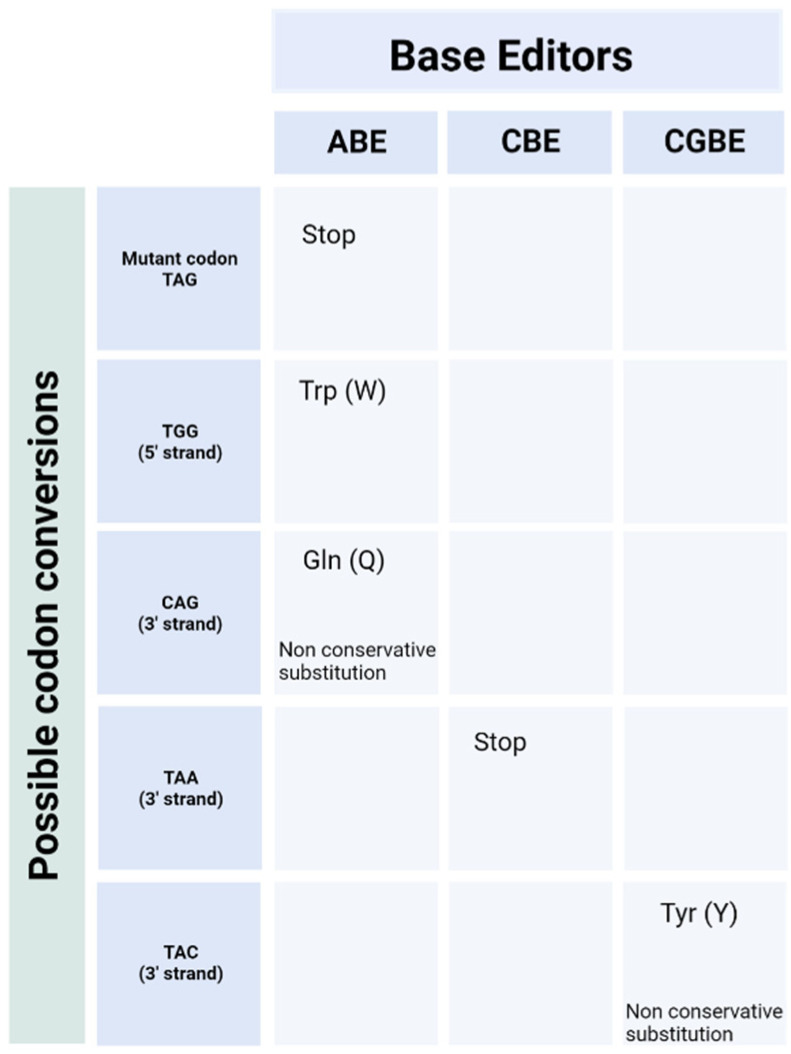
Amino acid conversions possible with base editing approaches to the c.11864G>A *USH2A* mutation. An adenine base editor can return the TAG stop codon back to the original TGG Typtophan codon. Adenine base editing of the A on the opposite strand of DNA or C to G base editing of the C on the opposite strand would enable read through, but results in non-conservative amino acid substitutions that could alter the structure of the protein (ABE) Adenine Base Editor, (CBE) cytidine base editor, (CGBE) C to G base editor Created with BioRender.com (accessed on 23 August 2022).

Base editors comprise a catalytically inactive Cas nuclease fused to a deaminase enzyme that mediates point mutations at specific locations. The two transition mutations mediated by cytidine and adenine base editors represent around 30% of human pathogenic variants [32]. A single transversion mutation that avoids cleavage of the DNA backbone is highly desirable for therapeutic use. Fry et al. found that 37.3% of pathogenic alleles in *USH2A* are correctable by base editing strategies [33]. The more common mutations in exon 13 of *USH2A* are not directly correctable by base editing, however adenine base editing of A to G on the translated strand returns the c.11864G>A TAG stop codon to the original TGG Tryptophan codon (Figure 4). Recently, the base editor VERVE 101 has entered phase 1b of clinical trial. VERVE 101 edits a single base to induce mis-splicing of the *PCSK9* gene and nonsense mediated decay of the mRNA transcript in heterozygous familial hypercholesterolaemia [34]. Musunuru et al. found that levels of low-density lipoprotein cholesterol were lowered by 60% in cynomolgus monkeys [35]. However, the base editing construct was delivered using lipid nanoparticles which have been found to transduce photoreceptors with poor effect [36] when compared to transduction in the liver. Cell specific promoters may improve levels of expression [37] as well as glutathione-targeted PEGylated liposomes [38]; however AAV particles remain the best transducers of photoreceptor cells at present, warranting smaller editing constructs.

#### 3.1.1. Smaller Proteins

**Figure 5:**
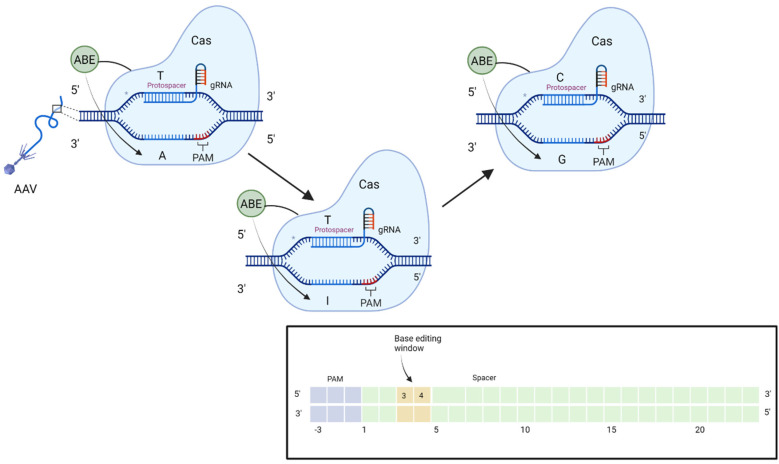
The base editing mechanism, which mediates an A to G conversion via an inosine intermediate. The CasMini protein has a maximal editing efficiency 3–4 base pairs in the 3′ direction to the PAM site. Created with BioRender.com (accessed on 18 August 2022).

Researchers are attempting to pair base editors with smaller Cas proteins for single vector packaging [39]. In silico analysis can search through billions of bacterial genomes to find organisms containing proteins with more desirable features. This high throughput evaluation has generated some successful results. The smallest Cas systems to date include CasMini (Cas12f/14) derived from uncultivated archaea ~40–70 kDa and CasPhi (Cas12j) derived from bacteriophages ~70–80 kDa [40]; halving the length of the more established Cas 9 or Cas12a systems ~1000–1500 amino acids [41]. Both CasMini and CasPhi offer real potential for single vector packaging of a base editor. Indeed, Xu et al. tethered ABE8e to the N terminus of CasMINI to create a construct that sits within the AAV packaging requirements [41]. Nonetheless, the more compact Cas systems often come with stiffer PAM sites. The 5′-NGG-3′ SpCas9 PAM site offers broad applicability. In contrast, some of the Cas12 PAMs (TTTV) and CasMINI PAM site (TTTR) have reduced value due to the requirement for multiple T nucleotides in target regions. Fused base editors also generate unique editing windows: the CasMINI editing window lies 3–4 base pairs downstream of the PAM site (Figure 5) and this creates additional constraints that render it inapplicable to *USH2A* and many other pathogenic mutations. CasPhi-2 has a more flexible PAM site (TBN) and could be paired with base editors to correct the W3955X mutation in *USH2A*: this represents a realistic option going forwards. Work will be needed to ascertain the base editing window of CasPhi-ABE and ensure bystander effects do not generate nonsense mutations.

#### 3.1.2. Truncating Existing Proteins

In tandem, scientists are working to truncate base editing enzymes for tethering to well-established Cas proteins. One of the most efficient base editing enzymes is ABE8e, comprised of two fused TadA domains. Richter et al. employed phage assisted continuous evolution (PACE) to enhance the catalytic rate of ABE7.10 by 590-fold to create ABE8e: the enhanced efficiency resulted in elevated off target effects, however these were negated by the introduction of a single point mutation (V106W) [42]. MiniABEmax consists of a single TadA domain; effectively halving the size of previous base editors. However, the truncation comes at the expense of efficiency, which is generally much lower and unlikely to provide therapeutic effect at this stage [43]. Additionally, a domain-inlaid SaCas9 ABE termed ‘MicroABE 1744’ has been incorporated within a single AAV vector [44]; however this construct also failed to reach therapeutic threshold. In future, the efficiency of these smaller base editors could perhaps be maximised via extension of the linkers [45] or editing of the most accessible nucleotides within the R-loop [46].

For CRISPR RNA based editing, which is limited to A>G edits, truncations of the RNA deaminase ADAR2 (ADAR2DD) can be paired with deactivated Cas13 for single vector packaging. It is possible that endogenous ADAR could enable single vector packaging, although it is doubtful whether this will be present at adequate levels. Small chemically modified oligonucleotides such as AIMers may need to be employed to direct endogenous ADAR to specific cellular RNA [47] and it is currently unknown whether this will be a viable strategy in photoreceptor cells. Cas systems that target RNA in a reversible manner have immense safety appeal, however the treatment would be temporary and development of an immune response with repeated injections is possible. Moreover, the Cas13 ADAR2DD generates bystander effects at adenine bases adjacent to the target adenine of the W3955X mutation. Future research could be directed towards minimising the base editing window, although this may reduce the scope of the construct.

### 3.2. Creation of a Smaller Prime Editor

Prime editing is a more recent development than base editing and has several advantages. Prime editing enables all possible base pair conversions; it demonstrates minimal indel occurrence and both bystander and off targets events are rare [48]. Additionally, prime editing is less constricted by PAM site availability close to the mutation. It would be possible to correct the c.2299del, c.2276G>T and c.11864G>A *USH2A* mutations with prime editing. Base editing currently shows higher levels of efficiency, however methods such as antisense pegRNAs, structurally modified pegRNAs and inhibition of mismatch repair have demonstrated improvements in prime editing activity [49,50,51]. It is a promising avenue to pursue, however the same problem with construct size exists.

#### 3.2.1. Smaller Proteins

For prime editing applications, small Cas9 nickase constructs are highly desirable. CjCas9 is the smallest variant to date and has shown promise in targeting genes in mouse retinal pigment epithelium [52], although studies suggest that the marginally larger SaCas9 orthologue demonstrates greater efficiency. However, Aird et al. evaluated delivery of a split *S. aureus* for prime editing and observed only ~0.5–1% rates of editing in vitro, possibly attributable to reduced residency time on the target DNA [53,54]. These smaller Cas9 orthologues also come at the expense of more stringent PAM sites. Hu et al. investigated smaller Cas9 orthologues such as SluCas9 and found this variant to be comparable to SaCas9 in size and efficiency: however, it is advantageous in maintaining the flexible NGG PAM site as opposed to the NRRGGT composition of SaCas9 [55]. Smaller Cas12 orthologues such as CasPhi could also be paired with reverse transcriptase enzymes. However, these would require modification for single stranded nicking as opposed to double strand cutting for safety optimisation, a difficult task considering both strands of the DNA are fed into the same cleavage site [40]. In addition, the design of the pegRNA would need to be adapted to the different cleavage mechanism of Cas12 proteins which cut downstream rather than upstream of the PAM site.

**Figure 6:**
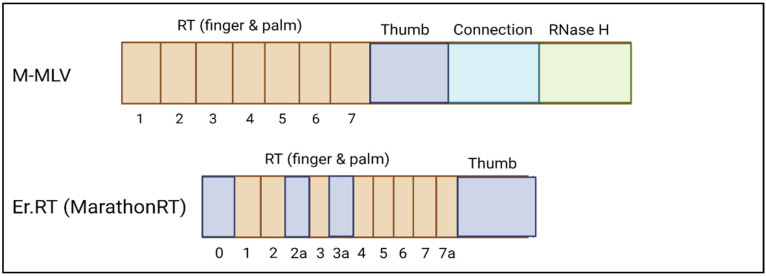
Comparison of the M-MLV and Er.RT structures, A Conceptual structure of the Murine Moloney Leukaemia virus reverse transcriptase with finger and palm domains, thumb, connection and RNase H domains. B One of the smallest reverse transcriptase enzymes derived from *Eubacterium rectale* that lacks the connection and Rnase H domains Created with BioRender.com (accessed on 23 August 2022).

The reverse transcriptase component of the prime editing construct represents another target. The reverse transcriptase enzyme mediates template directed correction of DNA mutations following single strand nicking. Liu et al. trialled two reverse transcriptase enzymes in combination with SpCas9 that are more compact in size than the standard Murine Moloney Leukaemia virus (M-MLV): a human codon-optimized *Eubacterium rectale* (*E.r*.) maturase reverse transcriptase (Figure 6) and GsI-IIC reverse transcriptase [24]. Both enzymes displayed editing activity confirming that alternative species of reverse transcriptase enzyme are compatible with prime editing, although efficiency did not exceed 5% in vitro. These reverse transcriptase enzymes would enable single vector packaging when paired with a smaller Cas9 orthologue such as SaCas9. It could be worthwhile to search for more compact reverse transcriptase enzymes derived from different species that generate higher levels of efficiency.

#### 3.2.2. Truncating Existing Proteins

Various attempts have been made to truncate SpCas9 with limited success. Based on the SpCas9 crystal structure, Nishimasu et al. found that deletion of the REC2 domain (encompassing residues 175–307) resulted in 50% loss of activity: it is whether this reduction in size offsets the significant reduction in activity [56]. Schmidt et al. employed gene family DNA shuffling to generate smaller synthetic RNA guided nucleotides (~1050 amino acids similar to SaCas9), while preserving the flexible NGG PAM site of SpCas9 and editing efficiency [57]. These new Cas9 constructs (such as sRGN3.1) could be promising and further work is required to explore their potential.

Several attempts have been made to truncate the M-MLV reverse transcriptase for packaging into a single AAV vector. Gao et al. exploited the modular structure of the enzyme, realising the dispensable role of the RNase H domain (used for RNA degradation in the RNA/DNA hybrid formed during synthesis of DNA) (Figure 6) [58]. They truncated the reverse transcriptase enough for packaging into dual vectors via a split intein approach. Zheng et al. also removed the RNase H domain, using the truncated construct to mediate codon insertion into the *Pcsk9* gene in mouse liver cells [59]. Further truncations into the connection domain also lead to a reverse transcriptase that retained a high percentage of activity [60]. The first 23 amino acids are not essential to the M-MLV function [61]. Nonetheless, further truncations are still required to circumvent the split intein method alongside pairing with a smaller Cas9 orthologue. It is quite possible that regions of the thumb, finger and palm domains of the enzyme are unnecessary for reverse transcription activity, and this is a future avenue to explore. Additionally, the RNAse H domains of other smaller reverse transcriptase enzymes could be removed to reduce the overall construct size.

## 4. Discussion and Future Directions

The AAV vector is a well-established delivery vehicle, demonstrating reduced immunogenicity and genomic integration when compared with other viral vectors [62]. There are numerous papers showing good therapeutic effect and minimal safety concerns and AAV vectors are now frequently used in clinical trial [63]. However, the 4.7 kb packaging limit means that it is not possible to fit the *USH2A* gene in its entirety into a single vector. Previous approaches have made use of CRISPR tools such as CRISPR-SKIP and CRISPR/Cas9 to edit the DNA directly. However, exon skipping often leaves the mRNA out of frame (as is the case for exon 61 of *USH2A*) and CRISPR/Cas9 systems induce double stranded breaks in the DNA. Base editing and prime editing tools afford much more specific correction of point mutations: base editors with inactivated nuclease activity leave the DNA strand intact and perform individual base transversions, whereas prime editors only nick one strand of the DNA and so have enhanced safety compared to previous techniques. However, the base editing or reverse transcriptase enzyme should be tethered to the Cas protein, increasing the payload of the CRISPR construct. Previously, dual vector approaches have been explored. However, editing efficiency is compromised and there are concerns over the immunogenicity of the bacterial derived inteins and ScFv-peptide fragments.

Future strategies for packaging base or prime editing machinery into a single AAV vector include the exploration of new organisms that harbour smaller proteins. Compact Cas proteins such as CasPhi could be paired with base editors to correct the W3955X mutation in *USH2A* and this represents a realistic option going forwards. Work will be needed to ascertain the base editing window of CasPhi-ABE and ensure bystander effects do not generate nonsense mutations. If a prime editing construct can be packaged within 4.7 kb then it represents a preferable option if efficiency can be maximised. Prime editing could correct all three of the commonest *USH2A* mutations due to its ability to mediate all four base pair transitions. It demonstrates fewer off target effects and has a decreased requirement for a flexible PAM site compared to base editing. A promising route for further exploration involves the smaller synthetic Cas9 constructs such as sRGN3.1 paired with smaller reverse transcriptase enzymes such as Marathon reverse transcriptase or truncated versions of M-MLV reverse transcriptase. An unexplored risk surrounds the potential of the reverse transcriptase to extend into the pegRNA scaffold beyond the primer sequence leading to unwanted insertions in the target site. Additionally, the exogenous reverse transcriptase activity harbours a risk of activating dormant pro-viral or retroviral elements. A long-term study may be pertinent to properly evaluate this safety concern. Nonetheless, if the prime editing construct can be packaged into a single AAV vector and generate therapeutic levels of editing this will ultimately be preferable to base editing due to its broad applicability. These new CRISPR tools represent an exciting prospect for treatment of mutations in *USH2A* and correction of currently untreatable blindness.

## Figures and Tables

**Table 1:** Usher syndrome subtypes, genes and proteins. https://www.institut-vision.org/en/28-diseases/91-usher-syndrom.html (accessed on 28 August 2022).

Subtype	Gene Locus	Gene Symbol	Length of cDNA (bp)	Protein Name	Protein Function/Possible Function
Usher 1A	Withdrawn				
Usher 1B	11q13.5	MYO7A	7363	Myosin VIIa	Actin-based motor protein
Usher 1C	11q15.1	USH1C	3241	Harmonin	PDZ-domain containing protein
Usher 1D	10q21-q22	CDH23	1750	Cadherin-23	Integral membrane adhesion protein
Usher 1E	21q21	Unknown	Unknown	Unknown	Unknown
Usher 1F	10q21.1	PCDH15	9135	Protocadherin15	Integral membrane adhesion protein
Usher 1G	17q24-25	USH1/SANS	3558	Sans	Putative scaffold protein
Usher 1H	15q22-23	Unknown	Unknown	Unknown	Unknown
Usher 2A	1q41	USH2A	18,938	Usherin	Integral membrane protein
Usher 2B	Withdrawn				
Usher 2C	5q14.3-21.3	VLGR1 (also known as GPR98)	19,557	Vlgr1	G-protein coupled receptor
Usher 2D	9q32-q34	WHRN (also know as DFNB31)	2889	Whirlin	PDZ-domain containing protein
Usher 3A	3q21-q25	USA3A	2259	Clarin 1	Integral membrane protein
Usher 3B	20q	Unknown	1828	Unknown	Unknown
